# An Analytical Framework for Optimizing the Renewable Energy Dimensioning of Green IoT Systems in Pipeline Monitoring

**DOI:** 10.3390/s25103137

**Published:** 2025-05-15

**Authors:** Godlove Suila Kuaban, Valery Nkemeni, Piotr Czekalski

**Affiliations:** 1 Institute of Theoretical and Applied Informatics, Polish Academy of Sciences, Baltycka 5, 44-100 Gliwice, Poland; 2 Department of Computer Engineering, Faculty of Engineering and Technology, University of Buea, Buea P.O. Box 63, Cameroon; nkemeni.valery@ubuea.cm; 3 Faculty of Automatic Control, Electronics and Computer Science, Silesian University of Technology, Akademicka 16, 44-100 Gliwice, Poland; piotr.czekalski@polsl.pl

**Keywords:** analytical and design framework, energy harvesting, green IoT (G-IoT) systems, energy storage, energy efficiency, energy leakage, pipeline monitoring

## Abstract

The increasing demand for sustainable and autonomous monitoring solutions in critical infrastructure has driven interest in Green Internet of Things (G-IoT) systems. This paper presents an analytical and experimental framework for designing energy-efficient, self-sustaining pipeline monitoring systems that leverage renewable energy harvesting and low-power operation techniques. We propose a hybrid approach combining solar energy harvesting with energy-saving strategies such as adaptive sensing, duty cycling, and distributed computing to extend the lifetime of IoT nodes without human intervention. Using real-world irradiance data and energy profiling from a prototype testbed, we analyze the impact of solar panel sizing, energy storage capacity, energy-saving strategies, and energy leakage on the energy balance of IoT nodes. The simulation results show that, with optimal dimensioning, harvested solar energy can sustain pipeline monitoring operations over multi-year periods, even under variable environmental conditions. We investigated the influence of design parameters such as duty cycling, solar panel area, the capacity of the energy storage system, and the energy leakage coefficient on energy performance metrics such as the autonomy or lifetime of the node (time required to drain all the stored energy), which is an important design object. This framework provides practical design insights for the scalable deployment of G-IoT systems in energy-constrained outdoor environments.

## 1. Introduction

Pipelines offer an efficient means of transporting fluids such as water, oil, and gas. However, leaks can lead to significant resource losses—especially in the case of water, which is becoming increasingly scarce—and can cause severe environmental contamination, particularly when hazardous substances like oil or gas are involved. A major challenge in municipal water distribution networks is leakage, which not only leads to significant water loss but also raises operational costs. If not promptly addressed, leaks can cause further deterioration of infrastructure [1]. Therefore, timely leak detection and rapid intervention are essential, highlighting the need for IoT-based monitoring systems along pipelines to identify and mitigate leaks efficiently. Advancements in technology, including the Internet of Things (IoT), Wireless Sensor Networks (WSNs), Artificial Intelligence (AI), distributed computing, and cloud computing, have made it possible to continuously monitor pipelines for leaks and corrosion.

A key challenge in deploying battery-powered sensor nodes for pipeline monitoring is their limited energy capacity, which requires frequent battery replacements. To address this issue, Green IoT solutions must be implemented, incorporating energy-efficient mechanisms and energy harvesting techniques to extend the operational lifespan of these sensors and reduce maintenance demands.

IoT devices typically rely on energy storage components such as batteries or supercapacitors, which are fully charged at deployment. Once the stored energy is entirely depleted, these components are replaced, leading to significant waste generation. Additionally, when the energy reserve is exhausted, the device remains inactive until its energy storage system is replaced. To mitigate waste and extend the operational lifespan of IoT devices (the duration before their energy reserves are fully drained), energy harvesting technologies are integrated. These systems collect energy from the environment, utilizing it to power the device while storing any surplus for future use.

A key approach in Green-IoT design is the implementation of energy-efficient strategies to enhance power management, thereby increasing device longevity while reducing e-waste and the carbon footprint associated with IoT deployments. Common energy-saving techniques include duty cycling, packet size reduction, transceiver optimization, energy-aware routing, and adaptive sensing. Additional methods involve minimizing protocol overhead, dynamic voltage and frequency scaling [2,3], and optimizing hardware and software for improved efficiency [3,4]. Furthermore, Green IoT embraces sustainable communication technologies such as Bluetooth, RFID, NFC, Zigbee, LoRa, and Sigfox, as well as energy-efficient architecture designs like green cloud computing, fog computing, and virtualization [5].

The authors of [6,7] explored an energy-saving strategy based on adaptive power management thresholds. When the energy level falls below a predefined limit, the device transitions into a low-power mode by adjusting operational parameters to minimize consumption. This may involve compromising Quality of Service (QoS) or reducing functionality to extend operational time.

Another core aspect of Green-IoT is energy harvesting, where devices extract power from ambient or external sources and either use it immediately or store it for later consumption. This approach not only prolongs device lifespan but also reduces the overall environmental impact of IoT networks. Ambient energy sources include radio frequency (RF) [8,9,10], photovoltaic (solar and artificial light) [11,12], thermal [13], and fluid-based sources (wind and hydro) [14]. External mechanical sources such as vibration, pressure, and strain, as well as human-based energy from physical movement or physiological activity, also contribute to energy harvesting in IoT systems [15].

Energy harvesting in IoT systems is inherently influenced by environmental variability, leading to fluctuations in energy availability that are often random and unpredictable [16]. To compensate for the unreliable and intermittent nature of renewable energy sources, storage systems—such as rechargeable batteries or supercapacitors—are integrated to retain excess energy for use during periods of low generation. This buffering capability is critical, as harvested energy may not always suffice to meet the continuous operational demands of the devices. Additionally, the energy consumption patterns of IoT nodes themselves can be uncertain and variable, exhibiting stochastic characteristics, as shown in the experimental data from [17]. As a result, designing efficient and sustainable Green IoT systems requires the careful co-optimization of three interdependent elements: the device’s power requirements, the harvesting potential of the chosen energy source, and the capacity and reliability of the storage component. Properly balancing these factors is essential to enhance energy efficiency, ensure high availability, and support long-term autonomous operation.

The primary goal in designing energy harvesting systems for IoT nodes is to appropriately size the harvesting and storage components to guarantee reliable energy availability and support long-term autonomous operation. Specifically, the system must be capable of storing enough energy to sustain the node throughout its intended lifetime—that is, from deployment until it becomes inoperative due to battery depletion and insufficient energy input. Over-dimensioning the energy harvesting and storage units can lead to increased costs and negatively affect critical design constraints such as device size, weight, portability, and overall environmental sustainability. On the other hand, under-dimensioning can compromise node functionality by raising the probability of energy shortages, thereby shortening the node’s lifespan and increasing service outages [18]. Consequently, accurate and practical modeling techniques are necessary to effectively determine the appropriate sizing and to evaluate the energy performance of harvesting, storage, and consumption subsystems within Green IoT architectures.

A widely adopted method for analyzing the energy performance of IoT nodes (without delving into the specific technicalities of energy harvesters, storage units, or node internals) is the use of discrete energy packet modeling. This concept, introduced by Gelenbe, involves representing energy harvesting, storage, and consumption as stochastic processes using energy packets [19,20,21]. Each energy packet corresponds to the minimum energy required for a basic operation, such as transmitting, receiving, or processing a data packet [22,23]. This modeling approach enables batteries and supercapacitors to be abstracted as energy queues, thereby allowing the application of queueing theory—traditionally used in network traffic analysis—to energy systems. It has been used extensively in the evaluation of self-sustaining IoT devices [24,25,26]. Alternatively, the dynamics of energy storage can be modeled through continuous-time stochastic frameworks such as fluid flow models [27,28,29] or diffusion processes [22,25,30,31], offering another lens for assessing energy behavior in Green IoT nodes.

An important consideration in dimensioning energy storage systems for IoT applications is energy leakage, which arises from the inherent imperfections and inefficiencies of storage devices. Accounting for such leakage complicates the modeling process, as it introduces an additional energy loss mechanism that operates alongside the usual energy harvesting and consumption processes. Notably, energy leakage is not an isolated factor—it is often dependent on the current energy level within the storage unit, making it a coupled process rather than an independent one. This interdependence adds complexity to system modeling and performance evaluation. Several studies have addressed this issue; for instance, refs [18,32] assume that the leakage rate is directly proportional to the amount of energy stored. Additionally, the leakage rate is sometimes assumed to grow exponentially with the amount of stored energy. Experimental analyses further support this assumption, with works such as [33,34,35] demonstrating that leakage in supercapacitors tends to grow exponentially with increasing amount of stored energy.

Only a limited number of studies have proposed analytical frameworks that adequately capture the practical challenges of dimensioning green IoT systems. In [16], the authors developed a probabilistic model-checking approach for sizing solar-powered wireless sensor nodes. Their framework examines how design variables—such as solar panel area, energy storage capacity, and device duty cycle—affect key reliability metrics, including the probability of energy depletion and system lifetime. Similarly, ref [36] introduced a Markov-based analytical model for solar-powered embedded systems using supercapacitors for energy storage. Their model incorporates energy leakage as an exponential function of the stored energy level. Both studies model solar energy generation as a discrete-time Markov process to capture the stochastic nature of the energy source.

Despite advancements in Green IoT system design, a structured IoT framework tailored to pipeline monitoring remains essential. This framework must optimize the sizing of energy harvesters and storage units while accounting for fluctuations in energy generation and consumption. The proposed framework also considers the imperfections of the energy storage system, such as energy leakage and charging and discharging inefficiencies. By doing so, researchers and engineers can assess different energy management strategies and protocols before real-world deployment.

This paper presents a comprehensive Green IoT (G-IoT) design framework for sustainable pipeline monitoring, focusing on the integration and optimization of energy consumption, harvesting, and storage. The proposed framework seeks to answer the following design questions. What are the optimal configurations of battery capacity and energy harvesting parameters to ensure uninterrupted IoT operation over long deployment periods? How do different environmental and system design factors (e.g., solar irradiance patterns and event occurrence rates) influence the energy sustainability and reliability of pipeline monitoring nodes? Moreover, how can energy-saving mechanisms (e.g., adaptive computing and duty cycling) improve the lifetime or autonomy of IoT nodes or ensure the long-term operation of IoT nodes? Additionally, how does energy leakage from the energy storage system influence the lifetime or autonomy of the IoT system? How can energy-aware design frameworks improve the operational lifetime and data fidelity of Green IoT systems used in pipeline infrastructure? The key contributions are as follows:

- We propose a comprehensive Green IoT (G-IoT) design framework for sustainable pipeline monitoring, focusing on the integration and optimization of energy consumption, harvesting, storage, and leakage;
- We analytically and empirically characterize the energy consumption of IoT nodes and quantify the impact of energy-efficient strategies such as adaptive sensing, duty cycling, and distributed computing on the autonomy of an IoT system;
- A photovoltaic-based energy harvesting model is developed and used to determine optimal solar panel sizing, incorporating real-world solar irradiance data and accounting for environmental and system losses;
- An IoT-based testbed is implemented to characterize the energy consumption pattern and power profile of an IoT system, which can then be used to simulate the long-term energy consumption of the energy system and be applied in the dimensioning of energy harvesting and storage systems for IoT-based pipeline monitoring;
- We investigated the influence of design parameters such as duty cycling, the area of a solar panel, the capacity of the energy storage system, and the energy leakage coefficient on energy performance metrics, such as the autonomy or lifetime of the node (time required to drain all the stored energy), which is an important design object.

## 2. Architectural Description

**Figure 1:**
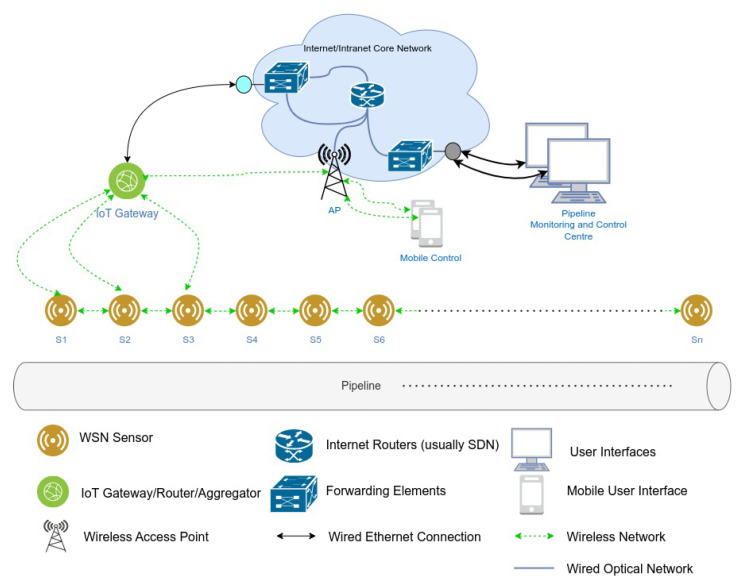
Linear wireless sensor network architecture for pipeline monitoring [37].

**Figure 2:**
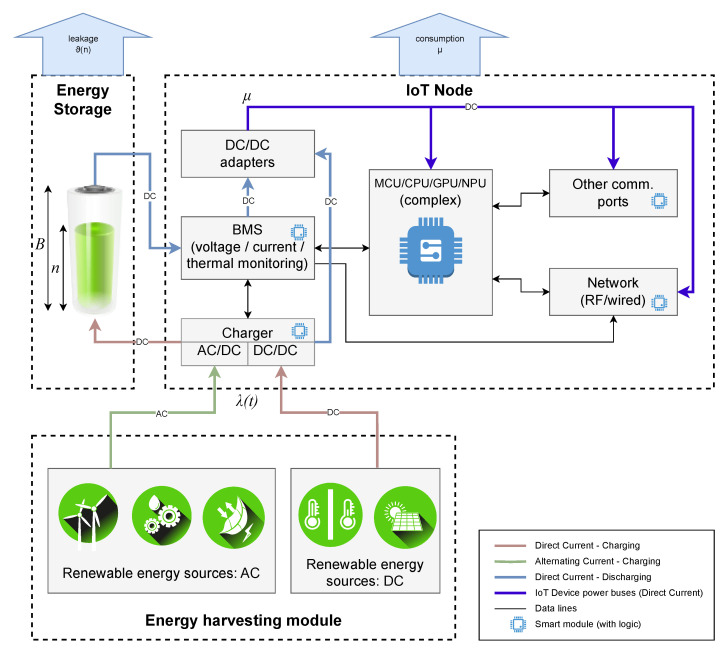
Architecture of a Green IoT node utilizing intermittent energy sources.

In IoT-based pipeline monitoring systems, sensor nodes are typically deployed along the pipeline in a linear topology, as illustrated in Figure 1. Each node in the system comprises three key components: an energy harvesting unit, an energy storage unit, and a sensor node, as shown in Figure 2. The harvested energy is stored in a battery or supercapacitor before being utilized by the sensor node in a harvest–store–consume configuration.

If the energy source generates alternating current (AC), such as wind, vibration, or radio frequency (RF) harvesters, an AC/DC converter is required to convert it to direct current (DC) for use by the sensor node. Conversely, if the energy is harvested in DC form, a DC/DC converter may be necessary to regulate the power according to the device and storage system requirements. An energy management system (e.g., a Battery Management System, BMS) is employed to optimize energy distribution and protect the system from power fluctuations.

The sensor node integrates a microcontroller unit (MCU), a wireless communication module, and sensors connected through designated ports. Accelerometers, for example, detect pipeline vibrations and compare them against predefined thresholds to identify potential leaks. The communication module enables data exchange either among sensor nodes or between the nodes and a gateway, depending on the deployment scenario.

Pipeline monitoring can generally be categorized into two approaches based on the data acquisition methodology:

- Regular sensing: The sensor node periodically wakes up at fixed intervals to collect and transmit data before returning to sleep. For instance, in a water distribution monitoring system [1], nodes typically activate twice a day under normal conditions or every 60 min if a potential leak is detected;
- Event-driven sensing: The sensor node remains in a low-power state and activates only when triggered by an anomaly, such as a sudden leakage event [17].

This adaptive monitoring strategy enhances energy efficiency while ensuring the timely detection of pipeline anomalies.

## 3. Sizing the Energy Consumption of the IoT Node

The power consumption of an IoT node can be sized using the average power consumption of the node in the active and sleep modes given in the datasheet of the specific device being considered. A more accurate approach is to estimate the power consumption of the device experimentally in the active and sleep modes. Then, the energy consumption of the node can be estimated depending on whether the sensing scenario is regular or event-driven. Below, we present an experimental and numerical approach to estimate the energy requirements of an IoT node for Water and Wastewater Pipeline Monitoring (WWPM) systems.

### 3.1. A Review of Energy-Efficient IoT Systems for Water and Wastewater Pipeline Monitoring (WWPM) Systems

Pipeline monitoring strategies are shaped by multiple factors, including communication protocols, assessment methodologies, power efficiency, monitoring techniques, sensor network connectivity, coverage, detection methods, and sensor types [38]. A key challenge in detecting leaks within Water and Wastewater Pipeline Monitoring (WWPM) systems using low-cost sensors is the potential for inaccurate leak signals. These inaccuracies often stem from the limited sensitivity of budget sensors and environmental noise interference, which can lead to false alarms. As a result, distinguishing genuine leak signals from noise remains a critical hurdle for leak-detection systems [39,40].

Current WWPM solutions face significant limitations, including high costs, excessive energy consumption, and delays in leak detection due to their centralized structure and reliance on intrusive sensors such as pressure and flow sensors. These sensors not only come with high installation costs but also require considerable power, making them inefficient for long-term deployment. Recently, the use of vibration sensors in WWPM systems has gained traction [40,41,42].

Vibration sensors provide a viable alternative for pipeline monitoring by exploiting the correlation between pressure variations in pipelines and surface vibrations. When a leak occurs, transient pressure changes generate localized increases in pipe surface acceleration along the pipeline [43]. Although this relationship is nonlinear, it remains monotonic, allowing vibration sensors to detect and pinpoint leaks [43,44]. Various types of vibration sensors, including accelerometers, piezoelectric transducers, and force-sensitive resistors, can be utilized for this purpose.

Compared to intrusive sensors, vibration-based monitoring systems offer easier installation, lower operational and maintenance costs, and reduced energy consumption. These advantages make them a cost-effective and energy-efficient alternative to traditional WWPM methods.

In many developing nations, water distribution systems predominantly rely on plastic piping. However, research indicates that leak-induced vibrations in plastic pipes do not travel long distances [45,46]. Consequently, reliable leak detection requires sensors to be placed in close proximity to one another to achieve higher spatial resolution [47]. High-precision accelerometers mounted on the pipe’s outer surface can effectively detect sudden increases in surface acceleration caused by leaks.

However, deploying such accelerometers necessitates reducing the spacing between sensors, significantly driving up costs and making large-scale implementation impractical, particularly in resource-constrained regions. To address this, low-cost MEMS accelerometers present a feasible alternative for affordable deployment. Nonetheless, these sensors come with limitations, including lower accuracy, which can hinder their ability to reliably differentiate leak signals from background noise [40,41,48,49]. Additionally, achieving real-time leak detection while ensuring prolonged system lifespan remains a challenge.

To address the need for cost-effective, real-time, energy-efficient, and sustainable pipeline monitoring, we propose Green IoT (G-IoT) strategies. These include distributed computing via a distributed Kalman filter, duty cycling enabled by interrupt-driven sleep/wake mechanisms, and adaptive sensing, which dynamically switches between low-power, low-accuracy sensors and high-power, high-accuracy sensors. Another energy-saving technique involves implementing energy-saving thresholds that trigger energy conservation modes once a predefined limit is reached, prolonging the system’s lifespan at the expense of some functionalities. The overarching goal is to develop a sustainable pipeline monitoring solution that balances cost efficiency, real-time performance, and reliability. Additionally, we provide an overview of energy consumption models for IoT nodes used in pipeline monitoring, considering both periodic sensing and event-driven sensing approaches.

### 3.2. Experimental Setup and Measurement Procedure

**Figure 3:**
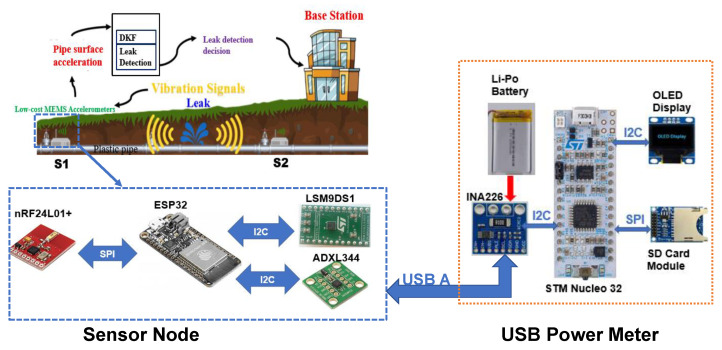
Architecture of the experimental testbed similar to the one used in [50].

The experimental study was carried out using a laboratory-based testbed featuring a high-pressure plastic pipeline, 12 m in length with a diameter of 25 mm. A control valve was placed at the midpoint of the pipeline to simulate leakage events. Two sensor nodes were deployed, positioned 2 m apart on either side of the valve, to monitor and capture data related to leak occurrences. A schematic of the experimental setup is illustrated in Figure 3.

**Figure 4:**
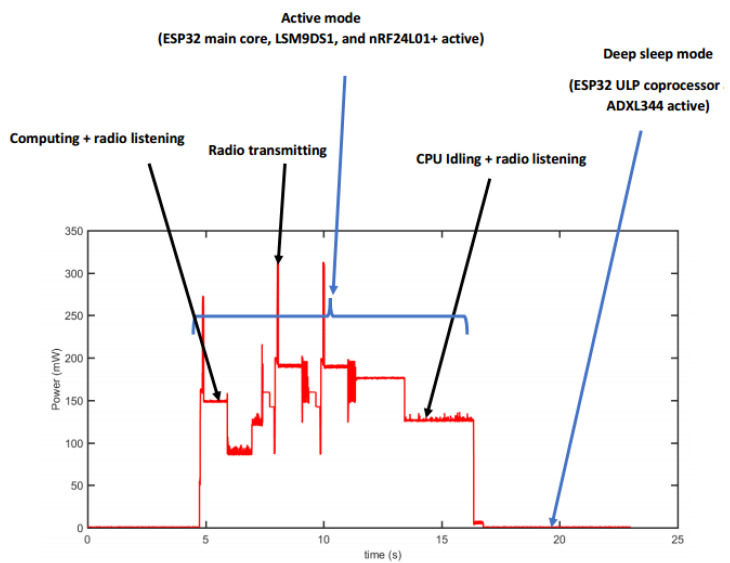
Power profile of the IoT node with duty cycling and adaptive sensing enabled. The fluctuations observed in the active mode are due to the sequential activation and deactivation of components (e.g., sensors, MCU, and transceivers) as the node transitions between functional states such as sensing, processing, transmitting, and receiving. Each state has a distinct energy profile, resulting in variations in power amplitude (from measurement).

**Figure 5:**
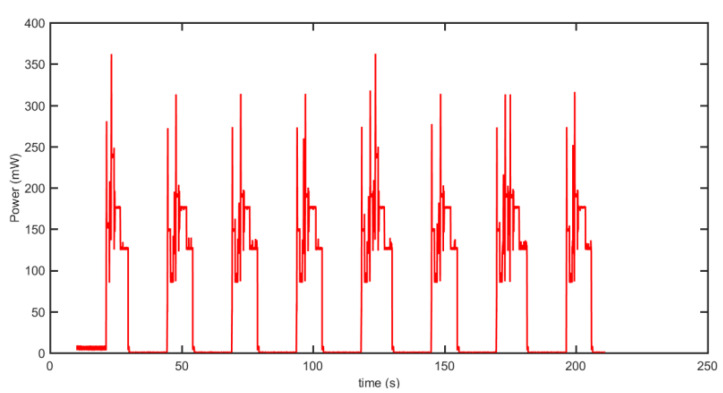
Power profile of the IoT node with duty cycling and adaptive sensing enabled, illustrating the power profile pattern for an IoT node with period or regular wake-up schedules (from measurement).

Each sensor node consists of multiple components: an ESP32 microcontroller unit (MCU) for processing, an nRF24L01+ transceiver for wireless communication, and two accelerometers—an LSM9DS1 high-power sensor, and an ADXL344 low-power sensor—for vibration detection. Power is supplied by a rechargeable 3.7 V 2000 mAh Li-Po battery. To monitor energy consumption, a USB power meter equipped with an INA226 module, interfaced with an STM Nucleo-32 F303K8 MCU, was employed. The power consumption data were displayed in real time and stored on an SD card throughout the experiment (refer to Figure 4 and Figure 5).

To achieve an energy-efficient and cost-effective real-time monitoring system, a hybrid energy management strategy was implemented. This approach integrates distributed computing, adaptive sensing, and duty cycling to optimize energy usage across various components, enhancing operational longevity while ensuring continuous monitoring.

A sleep/wake cycle mechanism was implemented to minimize power consumption and extend sensor node lifespan. This method deactivates energy-intensive components—including the MCU, transceiver, and sensors—during inactive periods. The ESP32 MCU, which typically consumes between 20 mA and 68 mA in active mode (modem sleep mode), remains in deep sleep mode whenever possible, reducing power draw to 150 µA. However, while in deep sleep, the node cannot detect pipeline events. To address this limitation, a low-power accelerometer continuously monitors the pipeline for anomalies. Upon detecting a potential leak, an interrupt is triggered to activate the other components, facilitating real-time event detection.

The large fluctuations in power amplitude during the active phase, as shown in Figure 4, are caused by internal transitions between various operational states of the sensor node. These include sensing, data processing, transmission, reception, and idle waiting. Each operation activates different hardware components, each with its own power requirement. For instance, the transceiver consumes significantly more power during data transmission than during idle listening, while the LSM9DS1 accelerometer consumes more energy than the low-power ADXL344. The transitions between these states, combined with the rapid on/off switching of components, result in the observed power variations.

The sensor node operates under the following sequence:

- At startup, the ESP32 MCU enters deep sleep mode, while the nRF24L01+ transceiver and LSM9DS1 accelerometer remain powered down. Meanwhile, the Ultra Low Power (ULP) coprocessor and ADXL344 accelerometer remain active. The ADXL344 continuously measures pipeline vibrations. If an acceleration surpasses the predefined threshold of 1.01 g, an interrupt is triggered, activating the remaining sensor components. If no significant vibration is detected, the system remains in its low-power state, with only the ULP coprocessor and ADXL344 sensor running;
- Upon activation, the LSM9DS1 accelerometer collects precise measurements, which the ESP32 MCU processes using the distributed Kalman filter (DKF) algorithm. The nRF24L01+ module facilitates data exchange between neighboring nodes for distributed data fusion;
- After aggregating sensor readings, the computed estimate is compared to a predefined baseline for leak detection. If the deviation exceeds a set threshold, an alarm is triggered before the system re-enters sleep mode. Otherwise, the node simply returns to its low-power state.

To assess the effectiveness of each energy conservation method, four experimental scenarios were conducted.

### 3.3. Power Consumption Analysis in IoT Nodes

In typical IoT deployments, an IoT node primarily operates in sleep mode and periodically wakes up to perform tasks such as sensing, processing, and communication. The wake-up behavior depends on whether the node follows a fixed sensing schedule or is triggered by events.

The total power required to operate an IoT node comprises several components: power for sensor measurements ($P_{\mathrm{sensing}}$), power consumed by the microcontroller unit (MCU) during computation ($P_{\mathrm{MCU}}$), power for transmitting ($P_{\mathrm{tx}}$) and receiving ($P_{\mathrm{rx}}$) data, power used by the system for real-time operating system (RTOS) tasks and management operations ($P_{\mathrm{system}}$), power consumed by the device in idle mode ($P_{\mathrm{idle}}$), and the power consumed in sleep mode ($P_{\mathrm{sleep}}$).

The overall power consumption of the node at any given time *t* is influenced by the duration spent in both active and sleep modes and can be expressed as

(1) $$P_{\mathrm{node}}\left(t\right)=D\cdot P_{\mathrm{active}}\left(t\right)+\left(1-D\right)\cdot P_{\mathrm{sleep}}\left(t\right)$$

Here, $P_{\mathrm{active}}$ denotes the total power consumed during active periods and is given by

(2) $$P_{\mathrm{active}}=P_{\mathrm{sensing}}+P_{\mathrm{MCU}}+P_{\mathrm{rx}}+P_{\mathrm{tx}}+P_{\mathrm{system}}+P_{\mathrm{idle}}$$

The duty cycle, denoted by *D*, is a critical parameter in optimizing power efficiency. It represents the fraction of time the node spends in active mode. A lower duty cycle—achieved by reducing active time and extending sleep durations—can substantially reduce overall energy consumption.

In water distribution monitoring systems, for example, IoT nodes periodically wake up to collect and transmit sensor data to edge or cloud platforms for further analysis. Prior studies [1] have shown that reducing the number of wake-ups per day increases sleep duration and reduces active time, thereby significantly lowering energy consumption.

The duty cycle is defined as

(3) $$D=\frac{T_{\mathrm{active}}}{T_{\mathrm{active}}+T_{\mathrm{sleep}}}$$

The total active time during a single wake-up cycle is given by

(4) $$T_{\mathrm{active}}^{i}=T_{\mathrm{sensing}}+T_{\mathrm{MCU}}+T_{\mathrm{rx}}+T_{\mathrm{tx}}+T_{\mathrm{system}}+T_{\mathrm{idle}}$$

where each *T* term corresponds to the duration spent in its respective operational state. During a complete operational period with $N_{\mathrm{wakeups}}$ number of wake-up events, the total active time is

(5) $$T_{\mathrm{active}}=N_{\mathrm{wakeups}}\cdot T_{\mathrm{active}}^{i}$$

The remainder of the time is spent in sleep mode, during which the node consumes significantly less power. The total energy consumption is, thus, directly influenced by the ratio of time spent in active vs. sleep modes. This depends on the wake-up pattern of the node, e.g., whether it is periodic or event-driven. Both scenarios are analyzed in the following sections.

#### 3.3.1. Power Consumption of IoT Node with Periodic Wake-Up Patterns

In periodic monitoring applications, an IoT device is configured to wake up at fixed, regular intervals—such as hourly, daily, weekly, or monthly intervals—to perform sensing, computation, and communication tasks before returning to sleep mode. The node remains in a low-power sleep state between scheduled wake-up events, minimizing energy consumption.

Let $\left\{T_{k}|T_{k}<T_{\mathrm{sim}}\right\}$ denote the set of wake-up times within the simulation period, where $T_{k}$ is the *k*-th wake-up time, and $T_{\mathrm{sim}}$ is the total simulation duration in hours. The condition $T_{k}<T_{\mathrm{sim}}$ ensures that wake-up events beyond the simulation window are excluded. Assuming the simulation spans $N_{\mathrm{days}}$ days and the node performs $n_{p}$ wake-ups per day, the complete set of wake-up times over the simulation period is given by

(6) $$t_{\mathrm{wake}}=\overset{N_{\mathrm{days}}-1}{\underset{d=0}{⋃}}\left\{t+24d|t\in \left\{T_{1},T_{2},\ldots ,T_{n_{p}}\right\}\right\}$$

For each $T_{k}\in t_{\mathrm{wake}}$, the device enters the active state for a fixed duration $T_{\mathrm{active}}$, during which it performs its assigned tasks. The node’s power consumption profile at time *t*, denoted by $P_{\mathrm{node}}\left(t\right)$, is modeled as

(7) $$P_{\mathrm{node}}\left(t\right)=\left\{\begin{array}{cc} P_{\mathrm{active}}, & \mathrm{if}\;t\in \left[T_{k},T_{k}+T_{\mathrm{active}}\right)\;\mathrm{for}\;\mathrm{any}\;T_{k}\in t_{\mathrm{wake}}\\ P_{\mathrm{sleep}}, & \mathrm{otherwise},\;\mathrm{for}\;t\in [0,T_{\mathrm{sim}}] \end{array}\right.$$

To provide a more detailed view of active mode behavior, we can decompose the active period into distinct operational phases: Sensing → Transmitting → Idle → Receiving → Sleeping. The power profile during each wake-up cycle can then be defined as

(8) $$P\left(t\right)=\left\{\begin{array}{cc} P_{\mathrm{sense}}, & \mathrm{if}\;T_{k}\leq t<T_{k}+T_{\mathrm{sensing}}\\ P_{\mathrm{transmit}}, & \mathrm{if}\;T_{k}+T_{\mathrm{sensing}}\leq t<T_{k}+T_{\mathrm{sensing}}+T_{\mathrm{tx}}\\ P_{\mathrm{idle}}, & \mathrm{if}\;T_{k}+T_{\mathrm{sensing}}+T_{\mathrm{tx}}\leq t<T_{k}+T_{\mathrm{sensing}}+T_{\mathrm{tx}}+T_{\mathrm{idle}}\\ P_{\mathrm{receive}}, & \mathrm{if}\;T_{k}+T_{\mathrm{sensing}}+T_{\mathrm{tx}}+T_{\mathrm{idle}}\leq t<T_{k}+T_{\mathrm{active}}\\ P_{\mathrm{sleep}}, & \mathrm{otherwise} \end{array}\right.$$

This function is evaluated for each scheduled wake-up $T_{k}$, and outside the active interval $\left[T_{k},T_{k}+T_{\mathrm{active}}\right)$, the device is assumed to remain in the sleep state with power consumption $P_{\mathrm{sleep}}$.

*Example: Twice-Daily Wake-Up Schedule*

Consider an IoT node that follows a fixed schedule, waking up twice a day at 6:00 AM and 6:00 PM (i.e., T={6,18}). Each wake-up cycle lasts for a duration $T_{\mathrm{active}}=6$ min, after which the node returns to sleep. The total active time per day is

(9) $$T_{\mathrm{daily}}=2\times T_{\mathrm{active}}.$$

Over a modeling period of $N_{\mathrm{days}}$, the cumulative active time becomes

(10) $$T_{\mathrm{total}}=N_{\mathrm{days}}\times T_{\mathrm{daily}}.$$

**Figure 6:**
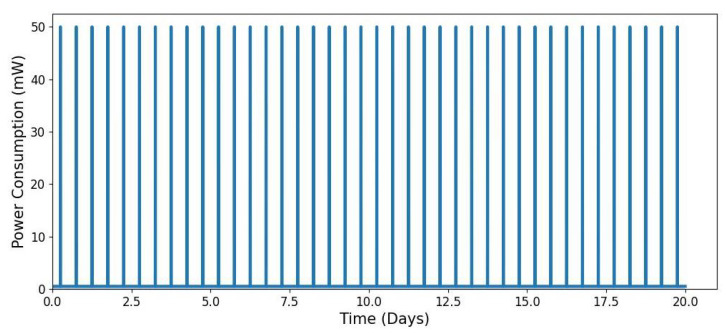
Periodic power consumption profile of an IoT node over a 20-day period.

**Figure 7:**
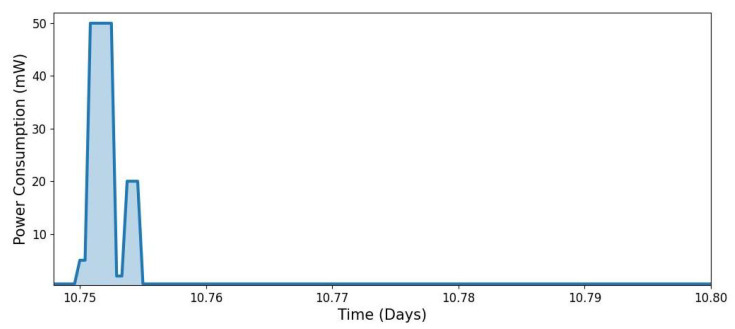
Snapshot of the power consumption profile, highlighting different operational states (Sleeping → Sensing → Transmitting → Idle → Receiving → Sleeping) (in the sequence from left to right).

Figure 6 and Figure 7 illustrate the power consumption profile of an IoT node that wakes up at 6 AM and 6 PM for sensor measurements and data transmission via a low-power communication channel.

The modelling was configured using the following parameters:

- Modelling time: number of days: $N_{\mathrm{days}}=20$, and total modelling time: $T_{sim}=24\times N_{\mathrm{days}}$;
- Time step resolution: Δt=0.01 h (equivalent to **36 s** per step);
- Power consumption levels: sleep mode: $P_{\mathrm{sleep}}=0.5$ mW, sensing mode: $P_{\mathrm{sensing}}=5$ mW, transmitting mode: $P_{\mathrm{tx}}=50$ mW, and receiving mode: $P_{\mathrm{rx}}=20$ mW;
- Wake-up cycle duration (6 min per cycle): Sensing: $T_{\mathrm{sensing}}=1$ min; Transmitting: $T_{\mathrm{tx}}=3$ min; Idle: $T_{\mathrm{idle}}=1$ min; Receiving: $T_{\mathrm{rx}}=2$ min.

Figure 6 exhibits a recurring pattern of power consumption spikes at 6 AM and 6 PM, corresponding to the scheduled wake-up intervals. These periodic bursts align with the expected operational schedule of the IoT node.

Additionally, Figure 7 provides a detailed view of the power levels associated with each operational state, demonstrating the distinct energy demands of sensing, transmitting, idling, and receiving. The observed power profile closely resembles the measured power consumption patterns of IoT nodes in real-world deployments, as reported in [51].

#### 3.3.2. Power Consumption of IoT Node with Random Event-Triggered Wake-Up Pattern

In event-driven IoT deployments, nodes remain in low-power sleep mode until an external trigger, specific events, or environmental stimuli occur, such as detecting a pipeline leak and then activating them. Upon activation, the node wakes up, collects sensor data, processes it, transmits or receives communication packets, and then returns to sleep. The intervals between consecutive wake-up events follow a Poisson process, meaning the time between events is exponentially distributed.

Let λ be the average wake-up rate per day and $\Delta t_{k}$ be the time between each pair of consecutive events i−1 and *i*, called the inter-arrival time. We assume that the inter-arrival time of events $\Delta t_{k}$ follows an exponential distribution. Then, the inter-arrival times $\Delta t_{k}$ are modeled as

(11) $$\Delta t_{k}∼\mathrm{Exponential}\left(\beta =\frac{24}{\lambda }\right),\;\mathrm{for}\;i=1,2,\ldots ,N_{wake}$$

where

$$\beta =\frac{1}{\lambda^{\prime }}\;\mathrm{is}\;\mathrm{the}\;\mathrm{average}\;\mathrm{time}\;\mathrm{between}\;\mathrm{events}\;\mathrm{in}\;\mathrm{hours}\;(\mathrm{mean}\;\mathrm{inter-arrival}\;\mathrm{time}),$$

$$\lambda^{\prime }=\frac{\lambda }{24}\;\mathrm{is}\;\mathrm{the}\;\mathrm{wake-up}\;\mathrm{rate}\;\mathrm{per}\;\mathrm{hour}.$$

The probability density function (PDF) of the interval times $\Delta t_{k}$ is

$$f\left(\Delta t_{k}\right)=\lambda^{\prime }e^{-\lambda^{\prime }\Delta t_{k}},\;\Delta t_{k}\geq 0$$

This implies that the device wakes up, on average, λ times per day, with an exponentially distributed gap between each pair of events. The wake times $T_{k}\in T=\left\{T_{1},T_{2},\ldots ,T_{N_{wake}}\right\}$ are computed by taking the cumulative sum of inter-arrival times:

$$T_{k}=\Delta t_{1}+\Delta t_{2}+\ldots \Delta t_{k}$$

The power profile of the node for each wake-up time $T_{k}$ is computed as in Equations (7) and (8) above for $k=1,2,\ldots N_{wake}$.

Let *N* be the random variable representing the number of wake-up events during the simulation duration $T_{sim}$. Since the wake-up events are assumed to follow a Poisson process, then

N∼Poisson(Λ)=Poisson(λ×T)

where Λ is the total wake-up rate over the interval $\left[0,\;T_{sim}\right]$. In this case, the expected number of wake-up events is

E[N]=λ×T

and the probability of observing *k* wake-up events over the interval $\left[0,T_{sim}\right]$ is

(12) $$P\left(N=k\right)=\frac{{\left(\lambda T\right)}^{k}}{k!}e^{-\lambda T},\;k=0,1,2,\ldots$$

The total energy consumption of the IoT node over the modelling period can be expressed as

(13) $$E_{\mathrm{total}}^{\mathrm{node}}=\sum_{i=1}^{N_{\mathrm{wake}}}E_{\mathrm{wake}}+E_{\mathrm{sleep}}$$

where $E_{\mathrm{total}}^{\mathrm{node}}$ is the total energy consumed over $N_{\mathrm{days}}$, $N_{\mathrm{wake}}$ is the total number of wake-up events, $E_{\mathrm{wake}}$ is the energy consumed per wake-up cycle, and $E_{\mathrm{sleep}}$ is the energy consumed in sleep mode.

During each activation, the node transitions through multiple power states. The energy consumed in each state is given by

(14) $$E_{\mathrm{state}}=P_{\mathrm{state}}\times T_{\mathrm{state}}$$

where $P_{\mathrm{state}}$ and $T_{\mathrm{state}}$ represent the power consumption and duration of each state, respectively. The total energy consumed per wake-up cycle is

(15) $$E_{\mathrm{wake}}=P_{\mathrm{sensing}}T_{\mathrm{sensing}}+P_{\mathrm{tx}}T_{\mathrm{tx}}+P_{\mathrm{idle}}T_{\mathrm{idle}}+P_{\mathrm{rx}}T_{\mathrm{rx}}$$

Since wake-up events follow a Poisson process, the expected number of wake-ups per day is $\lambda_{\mathrm{wake}}$. Over a modelling period of $N_{\mathrm{days}}$, the total energy consumed during active periods is

(16) $$E_{\mathrm{active},\;\mathrm{total}}=N_{\mathrm{wake}}\times E_{\mathrm{wake}}$$

When the node is inactive, it remains in sleep mode, consuming energy as

(17) $$E_{\mathrm{sleep}}=P_{\mathrm{sleep}}\times \left(T_{\mathrm{sim}}-N_{\mathrm{wake}}T_{\mathrm{active}}\right)$$

where $T_{\mathrm{sim}}=24\times N_{\mathrm{days}}$ represents the total simulation time, and $T_{\mathrm{active}}$ is the total duration of an active period.

Thus, the total energy consumption during the simulation period is

(18) $$\begin{array}{c} E_{\mathrm{total}}=N_{\mathrm{wake}}\left(P_{\mathrm{sensing}}T_{\mathrm{sensing}}+P_{\mathrm{tx}}T_{\mathrm{tx}}+P_{\mathrm{idle}}T_{\mathrm{idle}}+P_{\mathrm{rx}}T_{\mathrm{rx}}\right)\\ +P_{\mathrm{sleep}}\times \left(24N_{\mathrm{days}}-N_{\mathrm{wake}}T_{\mathrm{active}}\right) \end{array}$$

**Figure 8:**
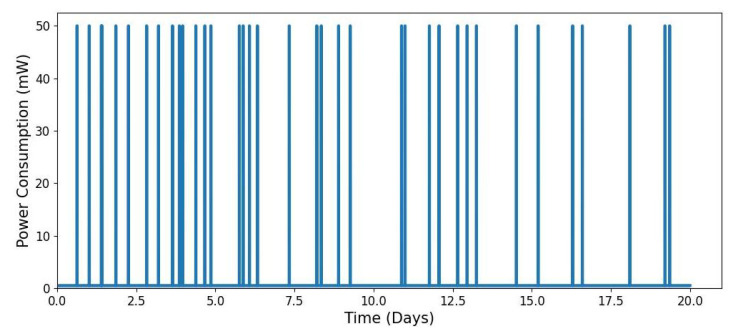
Power consumption profile of an event-driven IoT node over a 7-day period.

Figure 8 illustrates the power profile of an IoT node that wakes up based on event occurrences rather than fixed intervals. The modelling parameters are consistent with those used in Figure 6 and Figure 7, with an average wake-up rate of $\lambda_{\mathrm{wake}}=3$ events per day. Unlike the periodic wake-up pattern in Figure 6, the time between wake-ups is irregular, leading to fluctuating energy consumption. As event frequency increases, energy usage rises, whereas fewer events result in lower consumption. This variability introduces challenges in accurately predicting the node’s energy demand.

#### 3.3.3. Communication Energy Efficiency

The energy usage and operational lifespan of an IoT device are largely influenced by the hardware characteristics, communication standards, and the deployment environment. The device’s power consumption behavior, commonly referred to as the power profile, can be evaluated either through analytical modeling or real-world testing. The duration required to transmit a single IoT data packet depends on both its size and the channel capacity, and it is calculated as

(19) $$T_{tx}=\frac{m\cdot 8}{C},$$

where *m* represents the packet size in bytes, and *C* is the transmission rate in bits per second.

Based on Shannon’s capacity theorem, the energy ϵ required to transmit a packet of size *m* bytes using a transmission power $P_{tx}$ is expressed as [52]

(20) $$\epsilon =\left(\eta_{amp}\cdot P_{tx}+P_{0}\right)\cdot \frac{m\cdot 8}{W\log_{2}\left(1+SNR\right)},$$

Here, $\eta_{amp}$ denotes the efficiency of the power amplifier, $P_{0}$ corresponds to the constant circuit power overhead, and *W* is the available bandwidth. The signal-to-noise ratio (SNR) is calculated as

(21) $$SNR=\frac{P_{rx}}{P_{I}+P_{N}},$$

where $P_{rx}$ is the received signal power, $P_{I}$ represents the interference power, and $P_{N}$ is the noise power. As the transmitted signal propagates through the wireless medium, it experiences attenuation, and the received power is given by

(22) $$P_{rx}=P_{L}\cdot P_{tx},$$

with $P_{L}$ representing the path loss factor, as detailed in [52]. Consequently, the total transmission energy required to send $N_{T}$ packets is

(23) $$E_{\mathrm{total}}^{tx}=\sum_{i=1}^{N_{T}}\epsilon_{i}$$

Consider the following parameters for low-power IoT wireless communication:

- η=0.35—Power amplifier efficiency factor;
- $P_{0}=10\times 10^{-3}\;W$—Electronic circuit power;
- m=50bytes—IoT packet size;
- $W=1\times 10^{6}\;\mathrm{Hz}$—Channel bandwidth;
- $P_{I}=1\times 10^{-9}\;W$—Interference power;
- $P_{N}=1\times 10^{-9}\;W$—Noise power;
- $P_{L}=0.8$—Path loss factor.

**Figure 9:**
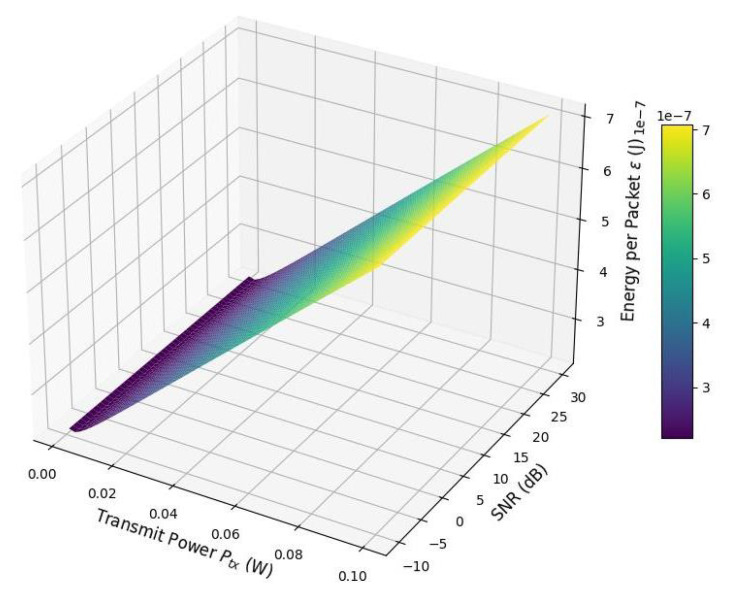
Energy per packet vs. transmit power and signal-to-noise ratio.

Figure 9 illustrates the relationship between the energy consumed per IoT packet (ϵ), transmit power ($P_{tx}$), and the signal-to-noise ratio (SNR). At low SNR levels (e.g., below 0 dB), the energy per packet is significantly higher due to poor channel conditions, which increase the time and energy required for successful transmission.

As the SNR improves, the channel capacity increases—thanks to the logarithmic dependence in Shannon’s formula—resulting in shorter transmission times and reduced energy consumption per packet. Initially, increasing $P_{tx}$ contributes to better SNR and lowers energy usage. However, beyond a certain threshold, further increases in $P_{tx}$ lead to higher energy consumption due to the added power required by the power amplifier and circuitry.

This behavior creates a distinct “valley” in the surface plot, representing an optimal operating region where the energy per packet is minimized. Operating outside this region—whether with too low or too high transmit power—results in inefficient energy usage.

For energy-constrained IoT applications, this reveals a critical trade-off between transmit power and communication reliability. Leveraging adaptive power control mechanisms can ensure that devices operate near this energy-optimal region, thereby extending battery life and improving system efficiency.

## 4. Dimensioning of the Energy Harvesting System

An energy harvesting system captures energy from various sources and converts it into electrical energy. This harvested energy is stored in an Energy Storage System (ESS) and then drained to supply the IoT node. Energy can be harvested from ambient sources such as solar radiation, photovoltaic cells, radio frequency (RF) waves, wind, hydro, and thermal sources. Additionally, mechanical energy from vibrations and pressure can be utilized.

The choice of an energy harvesting system depends on the energy demands of IoT nodes and the availability of suitable energy sources for a given application. IoT nodes deployed along long-distance pipelines can utilize solar, wind, flow, and vibration energy harvesters. However, the amount of energy harvested is typically small—ranging from a few hundred microwatts to a few hundred milliwatts—due to the limited size of the harvesters and the variability of available energy. Additionally, energy generation is intermittent, as it depends on fluctuating environmental conditions.

IoT devices operating at the fog and cloud layers are more power-intensive and require highly reliable energy sources, often in the few hundred Watts to a kilowatt range. Solar, wind, and pumped hydro systems are the most viable renewable energy sources for sustaining these high-demand nodes. However, since each renewable source has inherent limitations, hybrid energy systems are often employed. A combination of solar and wind energy has proven effective for powering off-grid cellular base stations, making it a promising solution for off-grid IoT base stations and other energy-demanding nodes within the fog and cloud layers.

This study models the energy generation of a solar panel under fluctuating weather conditions using a Markov chain approach. The total harvested energy is influenced by solar irradiance, weather states, temperature fluctuations, and panel efficiency. The dimensioning of the solar energy harvesting system involves the modelling and selection of the system parameters that influence the performance of the photovoltaic system and the generated output energy.

The photovoltaic (PV) system generates electricity by capturing solar energy and converting it directly into electrical energy through the photovoltaic effect [53]. The output power of a PV array can be estimated using the PV output power model developed by the National Renewable Energy Laboratory (NREL) [54], which was applied in [11,12] to size the solar capacity of base station sites. The power output at time *t* is given by

(24) $$P_{PV}\left(t\right)=A_{panel}\cdot P_{PV}^{*}\cdot \eta_{panel}\left(\frac{G(t)}{G^{*}}\right)\cdot f_{T}\left(t\right),$$

where the temperature-dependent correction factor is

(25) $$f_{T}\left(t\right)=\left[1+\vartheta \left(T_{amb}\left(t\right)+\frac{G(t)}{800}\left(NOCT-20\;{}^{\circ}C\right)-T_{PV}^{*}\right)\right].$$

In these equations, the following applies:

- $A_{panel}$ is the total area of the PV panels;
- $P_{PV}^{*}$ is the rated output power per unit area under Standard Test Conditions (STCs), as provided by the manufacturer;
- $\eta_{panel}$ is the energy conversion efficiency of the PV panels;
- $G^{*}$ is the solar irradiance under STC (typically $1000\;W/m^{2}$);
- G(t) is the solar irradiance at time *t*;
- ϑ is the power temperature coefficient (typically $-3\times 10^{-3}\left(1/{}^{\circ}C\right)$ for mono- and polycrystalline silicon [55]);
- $T_{PV}^{*}$ is the panel temperature under STCs (typically 25 °C);
- $T_{amb}\left(t\right)$ is the ambient temperature at time *t*;
- NOCT is the nominal operating cell temperature, typically provided by the manufacturer (typically 45 °C [54]).

### 4.1. Solar Irradiance Model

The daily variation in solar irradiance follows a sinusoidal pattern, peaking at midday and reaching zero at sunrise and sunset [56,57]:

(26) $$G\left(t\right)=G_{\max}\sin\left(\frac{\pi t}{L}\right)$$

where

- *t* represents the time in the sampling period;
- G(t) is the solar irradiance at time *t*;
- $G_{\max}$ is the peak irradiance from the previous day;
- *L* denotes the duration of daylight in sampling periods.

The instantaneous solar radiation received at a location throughout the day can be effectively modeled using a smooth sinusoidal function [57]:

(27) $$G_{\mathrm{solar}}\left(t\right)=G_{\mathrm{SC}}\times \max\left(\sin\left(\frac{\pi (t-t_{\mathrm{rise}})}{t_{\mathrm{set}}-t_{\mathrm{rise}}}\right),0\right)$$

where

- $G_{\mathrm{solar}}\left(t\right)$ is the instantaneous solar radiation (W/ $m^{2}$);
- $G_{\mathrm{SC}}=1361$ W/ $m^{2}$ is the solar constant;
- *t* is the time in hours;
- $t_{\mathrm{rise}}$ and $t_{\mathrm{set}}$ denote sunrise and sunset times, respectively.

This model captures the gradual increase and decrease in solar radiation from sunrise to sunset, peaking around solar noon. The argument of the sine function,

$$\frac{\pi (t-t_{\mathrm{rise}})}{t_{\mathrm{set}}-t_{\mathrm{rise}}},$$

maps the time interval from sunrise ($t=t_{\mathrm{rise}}$) to sunset ($t=t_{\mathrm{set}}$) onto the interval [0,π], ensuring the following:

- At sunrise ($t=t_{\mathrm{rise}}$), the sine term is zero;
- At solar noon ($t=\frac{t_{\mathrm{rise}}+t_{\mathrm{set}}}{2}$), the sine term reaches its maximum value of one;
- At sunset ($t=t_{\mathrm{set}}$), the sine term returns to zero.

The use of the max(…, 0) function ensures that solar radiation values remain non-negative, effectively modeling nighttime periods with zero irradiance.

Overall, this sinusoidal formulation offers a realistic and computationally efficient representation of daily solar radiation, adaptable to different seasons and geographical locations by adjusting $t_{\mathrm{rise}}$ and $t_{\mathrm{set}}$. A more realistic but complex model of solar irradiance can be found in [58].

### 4.2. Weather Modeling Using a Markov Chain

To capture the variability and stochastic nature of weather conditions, we employed a Markov chain framework to model transitions between distinct weather states. While prior studies, such as [59], have implemented a four-state Markov model, our approach simplifies the representation by using a three-state discrete-time Markov chain with the state space defined as

S={Sunny,Cloudy,Rainy}

The evolution of weather over time is described by a transition probability matrix, where each entry $p_{ij}$ indicates the likelihood of transitioning from state *i* to state *j* during a single time interval. The transition matrix is given by

(28) $$P=\left[\begin{array}{ccc} 0.7 & 0.3 & 0.0\\ 0.5 & 0.4 & 0.1\\ 0.2 & 0.3 & 0.5 \end{array}\right]$$

For example, the element $p_{12}=0.3$ implies a 30% probability of transitioning from a sunny to a cloudy condition in the next step. The matrix rows sum to 1, as required by the properties of a stochastic process.

Each weather state influences the effective solar irradiance reaching the photovoltaic (PV) panel. To incorporate this effect into the energy model, we define a weather-based irradiance attenuation factor $W_{\mathrm{eff}}$, which scales the solar input based on current weather conditions:

(29) $$W_{\mathrm{eff}}=\left\{\begin{array}{cc} 1.0 & (\mathrm{Sunny}:\;\mathrm{receives}\;100\%\;\mathrm{of}\;\mathrm{available}\;\mathrm{solar}\;\mathrm{radiation})\\ 0.6 & (\mathrm{Cloudy}:\;\mathrm{receives}\;60\%\;\mathrm{of}\;\mathrm{solar}\;\mathrm{radiation})\\ 0.3 & (\mathrm{Rainy}:\;\mathrm{receives}\;60\%\;\mathrm{of}\;\mathrm{solar}\;\mathrm{radiation}) \end{array}\right.$$

This simplified but effective Markov-based model allows us to dynamically adjust solar power generation based on probabilistic weather changes, providing a more realistic and adaptable simulation framework for energy yield prediction in variable weather effects. The adjusted solar radiation is then given by

(30) $$G_{\mathrm{adj}}\left(t\right)=G_{\mathrm{solar}}\left(t\right)\times W_{\mathrm{eff}}\left(t\right)$$

### 4.3. Temperature and Efficiency Degradation

The ambient temperature is assumed to follow a sinusoidal fluctuation throughout the day [60,61]. This variation can be modeled as

(31) $$T\left(t\right)=T_{a,\mathrm{mean}}+\Delta T\sin\left(\frac{\pi t}{24}\right)$$

where $T_{a,\mathrm{mean}}$ is the mean ambient temperature (e.g., $T_{a,\mathrm{mean}}=25\;{}^{\circ}C$), and $\Delta T=T_{a,\max}-T_{a,\mathrm{mean}}$ represents the amplitude of the daily temperature fluctuation. Here, $T_{a,\max}$ denotes the maximum daily temperature. For this study, we assume ΔT=5 °C, implying that the temperature oscillates between 20 °C and 30 °C.

The sinusoidal term $\frac{\pi t}{24}$ ensures a 24 h periodic cycle, capturing realistic day–night temperature transitions.

The panel efficiency is temperature-dependent, as efficiency typically decreases slightly with rising temperature. Assuming a linear relationship between temperature and efficiency [60,62], the efficiency degradation is modeled as

(32) $$\eta_{\mathrm{adj}}\left(t\right)=\eta_{\mathrm{panel}}\times \left(1-\vartheta (T\left(t\right)-T_{\mathrm{PV}}^{*})\right)$$

where ϑ is the temperature coefficient (or derating factor), which depends on PV technology. In this case, we assume β=0.003, meaning the efficiency decreases by 0.3% per degree Celsius above the reference temperature $T_{\mathrm{PV}}^{*}=25\;{}^{\circ}C$.

Here, $\eta_{\mathrm{panel}}$ is the nominal efficiency at 25 °C. If T(t)>25 °C, then $\left(T\left(t\right)-T_{\mathrm{PV}}^{*}\right)$ is positive, leading to reduced efficiency. Conversely, if T(t)<25 °C, the term becomes negative, and the panel efficiency improves slightly.

### 4.4. Energy Output Computation

From Equation (24), the instantaneous power output of the solar panel is determined by [58]

(33) $$P_{\mathrm{out}}\left(t\right)=G_{\mathrm{adj}}\left(t\right)\times A_{\mathrm{panel}}\times \eta_{\mathrm{adj}}\left(t\right)$$

where

- $A_{\mathrm{panel}}=0.001$$m^{2}$ (panel area);
- $\eta_{\mathrm{panel}}=0.18$ (nominal efficiency);
- $P_{\mathrm{out}}\left(t\right)$ is measured in Watts.

The total energy harvested over a given period *T* is computed as

(34) $$E_{\mathrm{total}}=\sum_{t=1}^{T}P_{\mathrm{out}}\left(t\right)\times \Delta t$$

where Δt=1 h.

**Figure 10:**
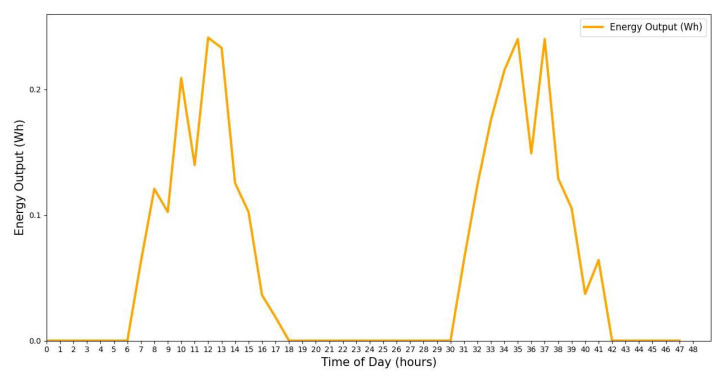
Simulated solar panel energy output over 2 days with Markov chain weather effects.

The preceding analysis offers a precise and adaptable assessment of solar energy harvesting, accounting for real-world fluctuations in sunlight, weather conditions, and temperature. Figure 10 illustrates the energy output variation of an IoT solar energy harvester. The power output starts increasing at sunrise (6 AM), reaches its peak at midday (12 PM), and decreases to zero by sunset (6 PM). This model provides a realistic estimation of energy generation by incorporating variations in sunlight, weather conditions, and temperature fluctuations.

## 5. Dimensioning the Energy Storage System

### 5.1. Modelling the Dynamics of the Energy Content of the Energy Storage Systems

The harvested energy is stored in an energy storage system and later utilized to power the IoT node. We assume that the sum of initially stored energy and harvested energy must always meet or exceed the energy demand of the IoT node [63], including the energy wasted due to energy leakage from the storage system. Thus, following the approach used in [63,64] while considering the possibility of energy leakage from the energy storage system, the continuous operation, the energy balance must satisfy the following condition:

(35) $$E\left[0\right]+\int_{0}^{t}P_{\mathrm{harvested}}\left(\tau \right)d\tau \geq \int_{0}^{t}P_{\mathrm{node}}\left(\tau \right)d\tau +\int_{0}^{t}P_{\mathrm{leakage}}\left(\tau \right)d\tau$$

where $P_{\mathrm{leakage}}$ represents the power dissipated due to energy leakage caused by non-ideal behaviors in the energy storage system. The performance of real-world energy storage systems in Internet of Things (IoT) and embedded devices is often degraded by various non-idealities. These include capacity fade over time, increased energy leakage, charge recovery effects, battery degradation due to repeated charge–discharge cycles, and charge redistribution [7]. These phenomena collectively impact the energy efficiency and lifespan of storage systems in practical applications.

The energy dynamics of an energy storage system in IoT environments can be accurately represented by accounting for both energy inflow and outflow processes. Energy inflow may originate from renewable sources such as solar harvesting or from wireless energy transfer mechanisms, while energy outflow arises due to device operations (e.g., sensing, processing, and communication) and inherent energy leakage caused by storage non-idealities.

To capture these dynamics, a fluid flow approximation is employed, where energy is treated analogously to a fluid entering and leaving a tank. This abstraction provides a continuous-time model that reflects the varying behavior of harvested energy and system demand.

The energy evolution over time is governed by the following differential equation:

(36) $$\frac{dE(t)}{dt}=P_{\mathrm{harvested}}\left(t\right)-P_{\mathrm{node}}\left(t\right)-L\left(E\left(t\right)\right),$$

subject to the physical storage constraint:

(37) $$0\leq E\left(t\right)\leq E_{\max},$$

where

- $P_{\mathrm{harvested}}\left(t\right)$ denotes the instantaneous energy arrival rate, which can be time-dependent, periodic (e.g., day/night solar patterns), or stochastic;
- $P_{\mathrm{node}}\left(t\right)$ represents the energy consumption rate, varying with the operational state and activity level of IoT devices;
- $P_{\mathrm{leakage}}\left(t\right)=L\left(E\left(t\right)\right)$ is the leakage rate that accounts for energy loss due to leakage or degradation effects. It is typically modeled as a proportional function of stored energy, such as L(E(t))=δE(t), where δ is the leakage coefficient (e.g., δ=0.001);
- $E_{\max}$ is the maximum energy storage capacity of the battery or supercapacitor.

Solving this differential equation analytically can be intractable due to the complexity and randomness of $P_{\mathrm{harvested}}\left(t\right)$, $P_{\mathrm{node}}\left(t\right)$, and $P_{\mathrm{leakage}}\left(t\right)$. As a result, numerical methods and simulations are commonly employed to evaluate system behavior, predict energy availability, and support energy-aware decision-making in real-time IoT operations.

Let $E_{\mathrm{harvested}}\left(t\right)$ and $E_{\mathrm{consumed}}\left(t\right)$ denote the energy harvested and consumed over the time interval [t,t+Δt]. The residual energy in the storage system at time *t* is represented as $E_{\mathrm{battery}}\left(t\right)$. The battery energy level is subject to

(38) $$0\leq E_{\mathrm{battery}}\left(t\right)\leq E_{\max}$$

While the previous analysis assumes an ideal energy storage system, real-world implementations—such as batteries and supercapacitors—exhibit non-ideal behaviors that degrade system efficiency over time. One of the most significant imperfections is energy leakage, which results in the gradual loss of stored energy, even when no load is applied. This phenomenon is especially pronounced in supercapacitors, where the leakage current often increases nonlinearly and, in some cases, exponentially with the stored energy level, as observed in empirical studies [7,33,34,35].

To account for these effects, the energy balance in the storage system (e.g., battery) over time can be described by the following discrete-time update equation:

(39) $$E_{\mathrm{battery}}\left(t\right)=\min\left\{E_{\max},\max\left\{0,E_{\mathrm{battery}}\left(t-\Delta t\right)+E_{\mathrm{harvested}}\left(t\right)-E_{\mathrm{consumed}}\left(t\right)\right\}\right\}$$

where

- $E_{\mathrm{battery}}\left(t\right)$ is the energy stored in the battery at time *t* (in mWh);
- $E_{\max}$ is the maximum energy storage capacity;
- $E_{\mathrm{harvested}}\left(t\right)$ is the energy harvested during time interval Δt;
- $E_{\mathrm{consumed}}\left(t\right)$ is the total energy consumed by the IoT device and lost due to leakage during the same interval.

The harvested energy from a solar panel can be estimated using

(40) $$E_{\mathrm{harvested}}\left(t\right)=G_{\mathrm{solar}}\left(t\right)\times A_{\mathrm{panel}}\times \eta_{\mathrm{panel}}\times \frac{\Delta t}{60}\times 1000$$

where

- $G_{\mathrm{solar}}\left(t\right)$ is the solar irradiance at time *t* (in Wh/ $m^{2}$);
- $A_{\mathrm{panel}}$ is the surface area of the solar panel (in $m^{2}$);
- $\eta_{\mathrm{panel}}$ is the energy conversion efficiency of the panel;
- Δt is the simulation time step (in minutes);
- A factor of 1000 converts Wh to mWh.

The total energy consumed by the IoT node is composed of the energy used for sensing, computation, wireless communication (transmission and reception), and internal leakage losses:

(41) $$E_{\mathrm{consumed}}\left(t\right)=P_{\mathrm{node}}\left(t\right)\times \frac{\Delta t}{60}+P_{\mathrm{leakage}}\left(t\right)\times \frac{\Delta t}{60}$$

where

- $P_{\mathrm{node}}\left(t\right)$ is the power consumption of the device at time *t*, which depends on its operational state (e.g., sleep, active, or transmit);
- $P_{\mathrm{leakage}}\left(t\right)$ represents the instantaneous power loss due to storage leakage, which may be modeled as a function of $E_{\mathrm{battery}}\left(t\right)$, such as $P_{\mathrm{leakage}}\left(t\right)=\delta \cdot E_{\mathrm{battery}}\left(t\right)$, where δ is a leakage rate coefficient.

### 5.2. Estimation of the Autonomy of the Device

To ensure uninterrupted device operation in the absence of energy harvesting, it is essential to estimate the node’s autonomy—the duration the system can sustain operation solely on stored energy. This autonomy is defined as

(42) $$T_{A}=\frac{Q_{b}\cdot DoD\cdot V_{b}}{P_{\mathrm{node}}}$$

For a given autonomy target $T_{A}$, the required rated battery capacity $Q_{b}$ (in mAh) can be estimated as

(43) $$Q_{b}=\frac{P_{node}}{DoD\cdot V_{b}}\cdot T_{A}$$

Here, $V_{b}$ is its nominal voltage, and DoD is the allowable depth-of-discharge. The total energy capacity $E_{\max}$ of the battery storage system is then computed as

(44) $$E_{\max}=Q_{b}\cdot V_{b}$$

An IoT system designer can extend the device’s lifetime (or autonomy, in the absence of energy harvesting) by adjusting key parameters such as the rated battery capacity $Q_{b}$ and reducing the node’s power consumption. Power consumption can be minimized through energy-efficient strategies, including optimizing the device’s duty cycle *D*, implementing adaptive sensing, applying dynamic voltage and frequency scaling (DVFS), and enhancing both hardware and software for low-power operation. This study investigates how device autonomy varies as a function of these tunable design parameters, offering insights into effective strategies for maximizing IoT node lifetime in energy-constrained environments.

Table 1 presents the effects of different energy-saving strategies on the power consumption and autonomy of an IoT node. In Experiment 1, only distributed computing is applied, resulting in high power consumption (125.42 mW) and a limited autonomy of 147.5 h. Introducing adaptive sensing in Experiment 2 slightly reduces the power consumption and marginally increases the lifetime. However, a significant improvement is observed in Experiment 3, where duty cycling reduces the power draw to just 2.48 mW, dramatically extending autonomy to over 7400 h. The best performance is achieved in Experiment 4, where all three strategies—distributed computing, adaptive sensing, and duty cycling—are combined, reducing power consumption to under 1 mW and extending autonomy to approximately 18,687 h. This demonstrates the critical role of combining multiple energy-saving techniques for enhancing the operational lifetime of IoT devices in energy-constrained environments.

Table 2 presents estimated autonomy values for selected battery types based on their nominal voltage and capacity. The calculation assumes the full discharge (DoD=1) and fixed power consumption of the IoT node, which include the power consumption of the various state of the device—e.g., sleep ($P_{\mathrm{sleep}}=0.01\;\mathrm{mW}$), sensing ($P_{\mathrm{sensing}}=5\;\mathrm{mW}$), and transmission ($P_{\mathrm{tx}}=50\;\mathrm{mW}$). We also assume that initially, the battery is charged to its full capacity. As expected, larger energy storage capacities significantly improve autonomy.

**Figure 11:**
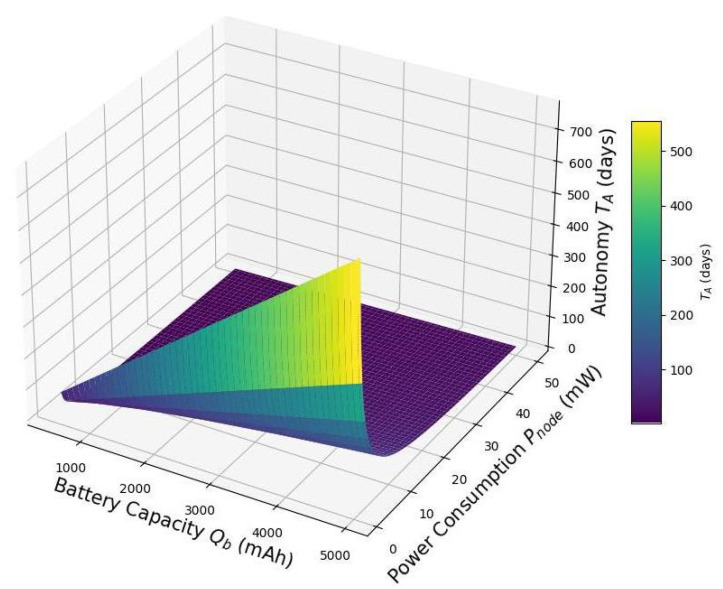
Battery autonomy $T_{A}$ vs. battery capacity $Q_{b}$ and node power consumption $P_{\mathrm{node}}$ for DoD=1 and $V_{b}=3.7\;V$.

Figure 11 visualizes the variation in node autonomy $T_{A}$ as a function of battery capacity $Q_{b}$ and power consumption $P_{\mathrm{node}}$ under fixed conditions (DoD=1, $V_{b}=3.7\;V$). The plot shows that autonomy increases linearly with battery capacity but decreases rapidly with increasing power demand. This underscores the critical role of energy efficiency in low-power designs: optimizing power consumption can yield greater autonomy gains than simply increasing battery size. These insights are essential for the design of sustainable, low-maintenance IoT deployments in energy-constrained environments.

**Figure 12:**
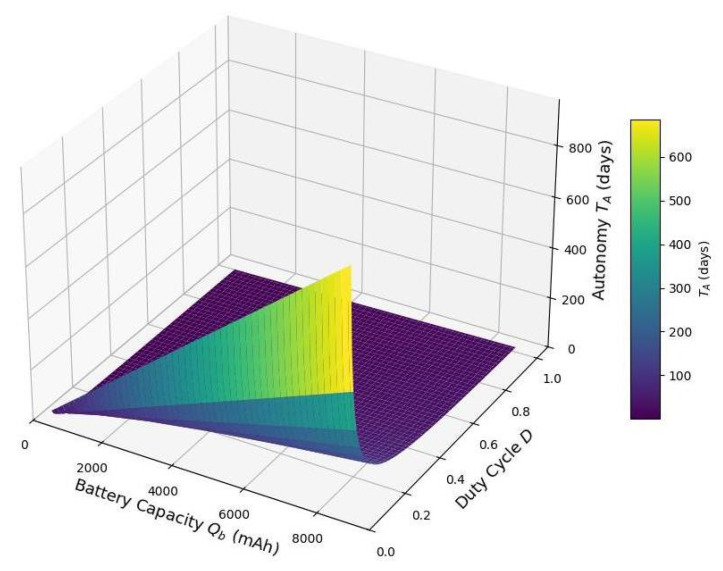
Battery autonomy $T_{A}$ vs. battery capacity $Q_{b}$ and duty cycle ((fraction of time active)) *D* for power in active mode, $P_{active}=50$ (mW), power in sleep mode (mW), $P_{sleep}=0.5$ (mW), DoD=1, and $V_{b}=3.7\;V$.

Figure 12 illustrates how the autonomy $T_{A}$, expressed in days, varies with both battery capacity $Q_{b}$ and duty cycle *D*. As expected and mentioned above, increasing the battery capacity results in a nearly linear increase in autonomy since more stored energy translates directly to a longer operational lifespan. Conversely, autonomy decreases as the duty cycle increases because higher duty cycles indicate that the device spends more time in its energy-intensive active state rather than its low-power sleep state. This leads to greater energy consumption, shortening the duration the device can operate without energy harvesting. The plot highlights the trade-off between node activity (duty cycle) and energy storage in determining system lifetime, emphasizing the importance of both low-power operation and adequate battery provisioning in IoT systems.

### 5.3. Energy Dynamics of the Storage System with Energy Harvesting

We address the dimensioning of the energy harvesting subsystem for a Green IoT node. Although the proposed analytical framework is designed to be general—applicable to various types of renewable energy sources—in this study, we specifically focus on photovoltaic (PV) energy harvesting. PV systems are not only simpler to design and implement but are also highly scalable and particularly well-suited for IoT deployments in outdoor environments.

For a photovoltaic energy harvesting system, the key design parameter that can be adjusted to ensure sustained operation of the IoT node is solar panel area. The mean daily harvested energy can be estimated using the expression [58]

(45) $${\mathrm{Energy}}_{\mathrm{harvested}/\mathrm{day}}=G_{\mathrm{avg}}\cdot A_{\mathrm{panel}}\cdot \eta_{\mathrm{panel}}\cdot H_{p}$$

Accordingly, the required solar panel area $A_{\mathrm{panel}}$ (in m^2^) to satisfy the node’s energy demand is given by

(46) $$A_{\mathrm{panel}}=\frac{E_{\mathrm{node}}}{H_{p}\cdot G_{\mathrm{avg}}\cdot TF\cdot \eta_{\mathrm{panel}}}$$

where $E_{\mathrm{node}}$ is the daily energy consumption of the IoT node (in Wh/day), $H_{p}$ denotes the peak sun hours per day at the deployment location (in hours/day), $G_{\mathrm{avg}}$ is the average solar irradiance (typically around 1000 W/m^2^ under standard test conditions), $\eta_{\mathrm{panel}}$ is solar panel efficiency (ranging from 0.15 to 0.22 for commercial panels), and TF is a tolerance factor accounting for system losses (commonly set to 1.4).

When dimensioning the renewable energy system for IoT applications, it is important to include a design margin of 20–30% to account for cloudy days, panel degradation, and conversion losses. Additionally, the solar panel should be appropriately sized to ensure that the energy storage system can be fully recharged within the available daylight hours.

**Figure 13:**
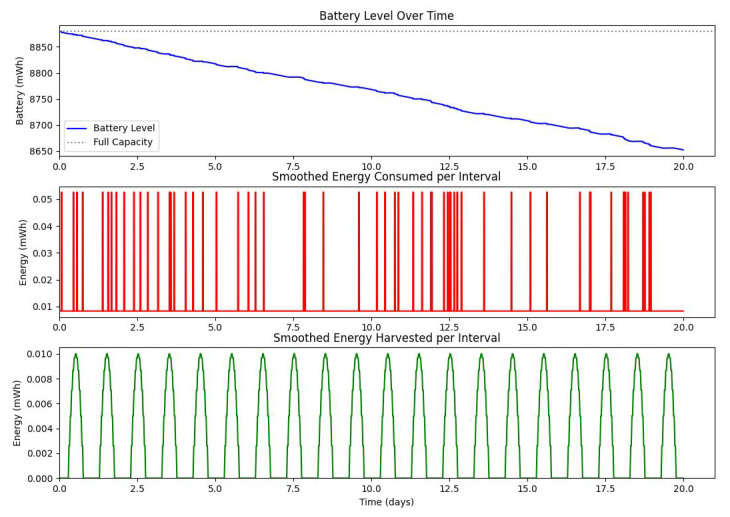
Dynamic evolution of harvested solar energy, stored battery energy, and energy consumption over a 20-day period, with a solar panel area of $A_{\mathrm{panel}}=0.5$ $m^{2}$ and a leakage coefficient of δ=0.001.

**Figure 14:**
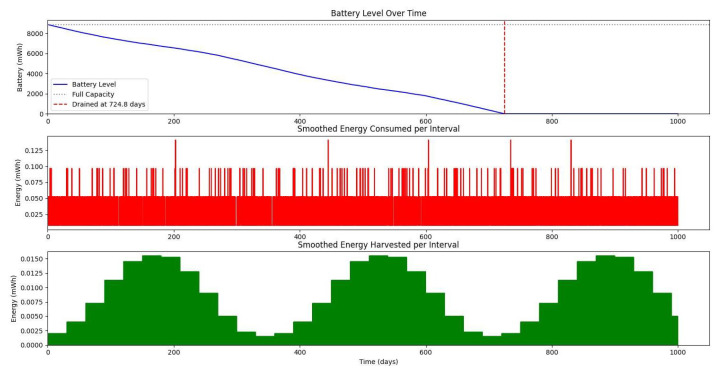
Dynamic evolution of harvested solar energy, stored battery energy, and energy consumption over a 1000-day period, with a solar panel area of $A_{\mathrm{panel}}=0.1$ $m^{2}$ and a leakage coefficient of δ=0.001.

**Figure 15:**
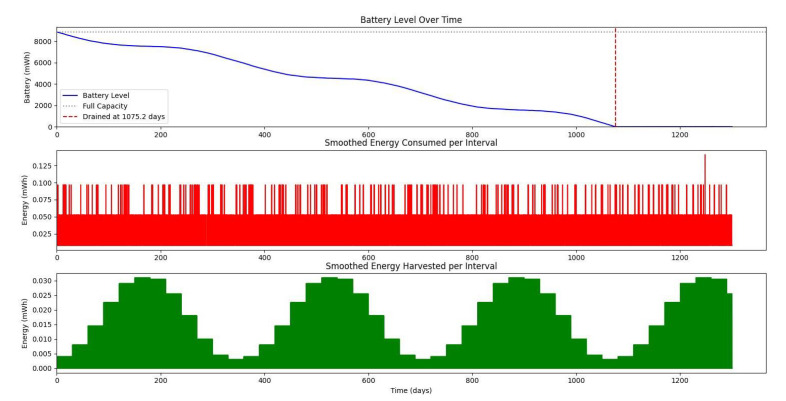
Dynamic evolution of harvested solar energy, stored battery energy, and energy consumption over a 1200-day period, with a solar panel area of $A_{\mathrm{panel}}=0.2$ $m^{2}$ and a leakage coefficient of δ=0.001.

**Figure 16:**
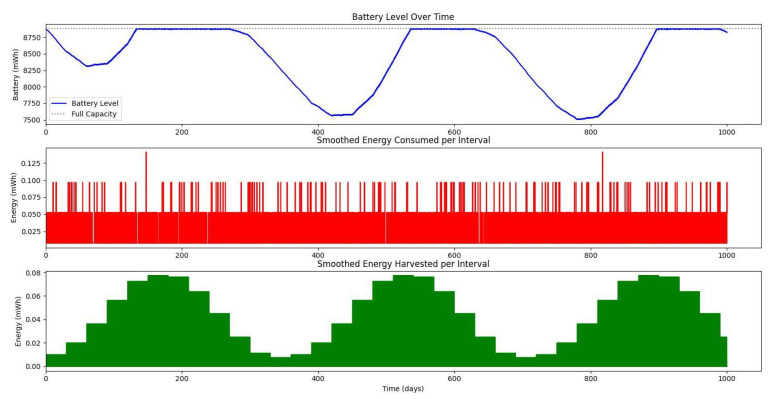
Dynamic evolution of harvested solar energy, stored battery energy, and energy consumption over a 1200-day period, with a solar panel area of $A_{\mathrm{panel}}=0.5$ $m^{2}$ and a leakage coefficient of δ=0.001.

**Figure 17:**
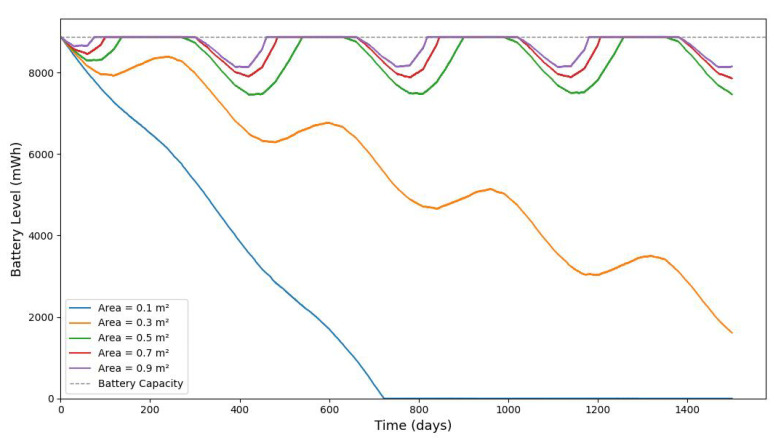
The evolution of stored battery energy over a 1500-day period for various solar panel areas of $A_{\mathrm{panel}}(m^{2})$ and a leakage coefficient of δ=0.001.

**Figure 18:**
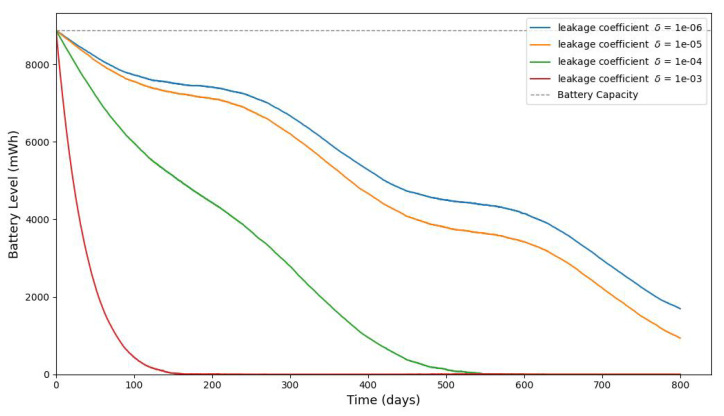
The evolution of stored battery energy over a 1500-day period for various leakage coefficients of δ and a solar panel area of $A_{\mathrm{panel}}=0.2\;m^{2}$.

**Figure 19:**
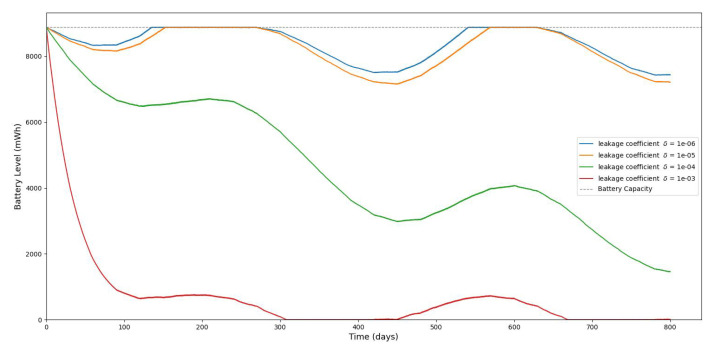
The evolution of stored battery energy over a 1500-day period for various leakage coefficients of δ and a solar panel area of $A_{\mathrm{panel}}=0.5\;m^{2}$.

Figure 13, Figure 14, Figure 15, Figure 16, Figure 17, Figure 18 and Figure 19 illustrate the influence of solar irradiance, solar panel area, and energy leakage on the dynamic behavior of energy harvesting and storage in solar-powered IoT systems, specifically for pipeline monitoring in Bydgoszcz, Poland.

**Figure 20:**
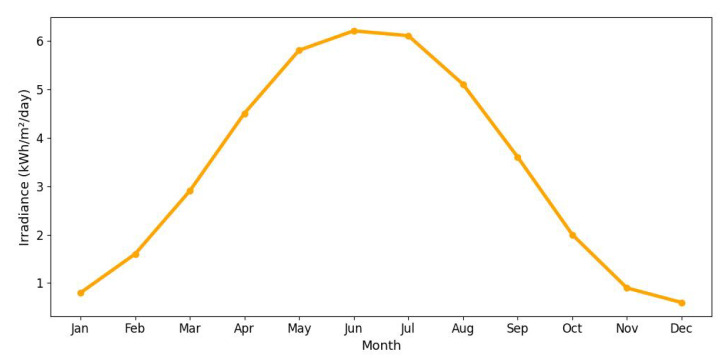
Average monthly peak solar irradiance (in Bydgoszcz, Poland) over one year. The irradiance increases from winter to summer months, reaching a maximum around June, and then declines, indicating the influence of seasonal changes on solar energy availability. Data obtained from [65].

Figure 20 shows the average monthly solar irradiance, which peaks around June and declines toward winter, highlighting the seasonal nature of solar energy availability. Figure 13, Figure 14, Figure 15 and Figure 16 illustrate the time evolution of harvested energy, stored battery energy, and energy consumption. For a short-term 20-day period (Figure 13), a solar panel with an area of $A_{\mathrm{panel}}=0.5\;m^{2}$ effectively sustains battery levels despite leakage, demonstrating that the harvested energy offsets the device’s consumption. In contrast, Figure 14 and Figure 15 show that smaller panel areas ($A_{\mathrm{panel}}=0.1$ and $0.2\;m^{2}$) fail to provide sufficient energy in long-term deployments, resulting in battery depletion. In the case of $A_{\mathrm{panel}}=0.1\;m^{2}$, the stored energy in the battery drained after 724.84 days, and in the case of $A_{\mathrm{panel}}=0.2\;m^{2}$, the time required to drain all the stored energy is 1075.19 days. When the panel area is increased to $0.5\;m^{2}$ over a 1200-day period (Figure 16), the system maintains consistent battery levels, ensuring reliable operation.

Figure 17, Figure 18 and Figure 19 serve to further evaluate the impact of panel area and leakage coefficient on long-term battery dynamics. Since some of the stored energy leaks out of the storage system and is wasted, shortening the time required to deplete all the stored energy, we investigate the influence of leakage on the dynamic evolution of the stored energy. Figure 17 shows that larger panel areas sustain higher energy storage over 1500 days. Figure 18 and Figure 19 reveal that increased leakage coefficients reduce energy retention, even with larger panels, highlighting the need for low-leakage energy storage solutions in long-duration IoT deployments. In this paper, we assume that the energy leak rate is proportional to the amount of stored energy, as discussed in [18,32]. In some energy storage systems, the leakage rate increases exponentially with the amount of stored energy, as in the case of supercapacitors shown in [33,34,35].

**Figure 21:**
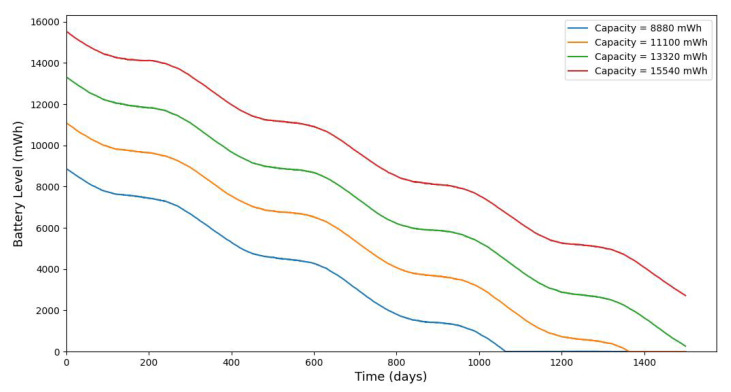
The evolution of stored battery energy over a 1500-day period for various battery energy capacities $E_{max}=Q_{b}\cdot v$ and a solar panel area of $A_{\mathrm{panel}}=0.2\;m^{2}$, $Q_{b}=\left\{2400,3000,3600,4200\right\}$, v=3.7, and δ=0.001.

**Figure 22:**
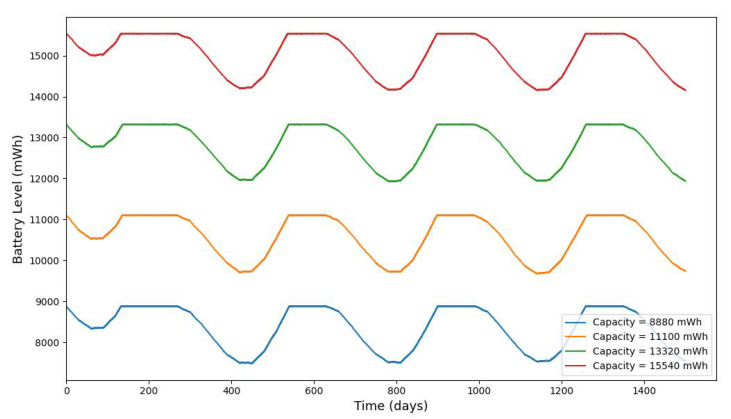
The evolution of stored battery energy over a 1500-day period for various battery energy capacities $E_{max}=Q_{b}\cdot v$ and a solar panel area of $A_{\mathrm{panel}}=0.5\;m^{2}$, $Q_{b}=\left\{2400,3000,3600,4200\right\}$, v=3.7, and δ=0.001.

Figure 21 and Figure 22 illustrate how varying battery capacities influence the sustainability and stability of an energy harvesting IoT device over long-term deployment (1500 days). As expected, devices with larger battery capacities exhibit higher and more stable battery levels over time. This is because larger batteries can store more harvested energy during periods of excess solar irradiance, which helps buffer against periods of low energy availability (e.g., during winter months or consecutive cloudy days). In contrast, smaller batteries reach their capacity limit more quickly and may waste excess harvested energy while also being more prone to depletion during energy-scarce periods.

The simulation also demonstrates the trade-off between storage size, solar panel area, and energy reliability. Although increasing the battery capacity does not improve the energy harvesting rate, it significantly reduces the risk of battery depletion and ensures smoother operation with fewer interruptions. These findings support the importance of optimizing battery and solar panel sizing in conjunction with expected power consumption and harvesting profiles to ensure the continuous operation of IoT systems, particularly in energy-constrained or remote applications like pipeline monitoring.

## 6. Conclusions and Future Work

This paper introduces a comprehensive Green IoT (G-IoT) framework tailored to sustainable and energy-efficient pipeline monitoring. To address critical challenges such as limited battery autonomy, high operational costs, and the demand for timely leak detection, we integrated a suite of energy-efficient strategies—including adaptive sensing, duty cycling, and distributed computing—with renewable energy harvesting from solar sources. The proposed approach not only minimizes power consumption but also leverages environmental energy to support the long-term, autonomous operation of IoT nodes.

Our experimental evaluations and detailed simulations demonstrate the use of the proposed framework for the dimensioning of Green IoT systems for pipeline monitoring, although the framework is not limited to the design of G-IoT systems for pipeline monitoring. The results reveal that with appropriately dimensioned solar panels and an energy-aware design, the IoT nodes can achieve energy self-sufficiency over extended periods, even under realistic irradiance and leakage conditions. These findings demonstrate the potential of our G-IoT framework to enable scalable, maintenance-free, and reliable monitoring in critical infrastructure systems such as pipelines.

Future research can further refine this framework by exploring advanced machine learning models for intelligent energy management, enhancing energy prediction accuracy, and integrating additional energy harvesting modalities. The development of ultra-low-power hardware and communication protocols can further improve the efficiency of Green IoT solutions. Ultimately, the proposed framework contributes to the advancement of sustainable pipeline monitoring, minimizing environmental impact while ensuring resource efficiency and infrastructure integrity.

Although the ESP32 microcontroller was selected for our testbed due to its dual-core processing capabilities and support for on-node distributed algorithms (e.g., Kalman filtering), it is not optimized for ultra-low-power operation during deep sleep. The measured sleep current of 150 μA is significantly higher than what is typical in energy-constrained sensor applications, where currents below 1 μA are common.

Future designs could improve energy efficiency by replacing an ESP32 with a low-power microcontroller and integrating a wake-up controller, such as the TI TPL5111 or NXP PCF2123. These components can reduce standby current to the nanoampere range by employing a CMOS switch to disconnect high-power subsystems during idle periods. Incorporating such hardware-level energy optimization would greatly enhance battery life, particularly in event-driven sensing scenarios characterized by long idle durations.

## Figures and Tables

**Table 1:** Effect of energy-saving strategies on IoT node power consumption and autonomy, based on measured data from the experimental setup in Section 2.

Experiment	Configured Energy-Saving Mechanism	$P_{\mathrm{node}}$ (mW)	$T_{A}$ (h)
1	Distributed Computing only	125.42	147.50
2	Distributed Computing + Adaptive Sensing	123.76	149.48
3	Distributed Computing + Duty Cycling	2.48	7459.68
4	Distributed Computing + Adaptive Sensing + Duty Cycling	0.99	18,686.87

**Table 2:** Battery specifications and estimated life without energy harvesting (device autonomy).

Battery Type	Capacity $Q_{b}$ (mAh)	Voltage $V_{b}$ (V)	Autonomy $T_{A}$ (Days)
AA Alkaline	2500	1.5	2100.0
Li-Ion 18650	3000	3.7	6216.0

## Data Availability

All the data used and created in the research studies are presented in the paper.
